# Dichloridobis[4,4,5,5-tetra­methyl-2-(5-methyl-1*H*-imidazol-4-yl-κ*N* ^3^)-2-imidazoline-1-oxyl 3-oxide-κ*O*]copper(II)

**DOI:** 10.1107/S1600536811040475

**Published:** 2011-10-08

**Authors:** Zhi Yong Gao, Wen Bei Zhang

**Affiliations:** a College of Chemistry and Environmental Science, Henan Normal University, Xinxiang 453002, People’s Republic of China

## Abstract

In the title complex, [CuCl_2_(C_11_H_17_N_4_O_2_)_2_], the Cu^II^ ion, lying on an inversion center, is six-coordinated in a distorted octa­hedral [Cu(N_2_O_2_Cl_2_)] environment by two *N*,*O*-bidentate nitronyl nitroxide radical ligands and two *trans*-chloride anions. In the imidazoline-1-oxyl-3-oxide unit of the ligand, the four methyl groups and the C atoms to which they are bonded are disordered over two sets of sites, with a refined occupancy ratio of 0.737 (5):0.263 (5).

## Related literature

For general background to mol­ecular magnetic materials, see: Stroh *et al.* (2003 ▶); Kahn *et al.* (2000 ▶); Fursova *et al.* (2003 ▶). For complexes including nitronyl nitroxide ligands, see: Muppidi & Pal (2006 ▶); Wang *et al.* (2005 ▶); Gao *et al.* (2010 ▶). For the synthesis of the ligand of the title complex, see: Ullman *et al.* (1970 ▶, 1972 ▶).
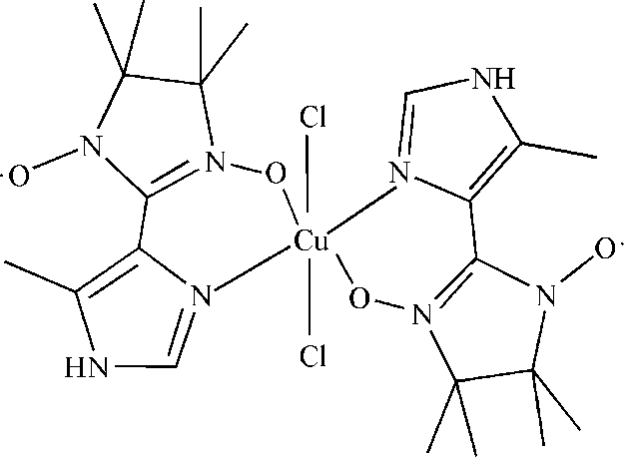


## Experimental

### 

#### Crystal data

[CuCl_2_(C_11_H_17_N_4_O_2_)_2_]
*M* *_r_* = 609.01
Monoclinic, 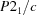

*a* = 7.9966 (10) Å
*b* = 14.1981 (18) Å
*c* = 12.9266 (17) Å
β = 94.169 (3)°
*V* = 1463.8 (3) Å^3^
*Z* = 2
Mo *K*α radiation
μ = 0.97 mm^−1^
*T* = 295 K
0.43 × 0.27 × 0.17 mm

#### Data collection

Bruker SMART APEXII CCD area-detector diffractometer
Absorption correction: multi-scan (*SADABS*; Bruker, 2002 ▶) *T*_min_ = 0.682, *T*_max_ = 0.855
12447 measured reflections
3359 independent reflections
2607 reflections with *I* > 2σ(*I*)
*R*_int_ = 0.027

#### Refinement

*R*[*F* ^2^ > 2σ(*F* ^2^)] = 0.060
*wR*(*F* ^2^) = 0.202
*S* = 1.06
3359 reflections
138 parameters
63 restraints
H-atom parameters constrained
Δρ_max_ = 1.09 e Å^−3^
Δρ_min_ = −1.07 e Å^−3^

### 

Data collection: *SMART* (Bruker, 2002 ▶); cell refinement: *SAINT* (Bruker, 2002 ▶); data reduction: *SAINT*; program(s) used to solve structure: *SHELXS97* (Sheldrick, 2008 ▶); program(s) used to refine structure: *SHELXL97* (Sheldrick, 2008 ▶); molecular graphics: *SHELXTL* (Sheldrick, 2008 ▶); software used to prepare material for publication: *publCIF* (Westrip, 2010 ▶).

## Supplementary Material

Crystal structure: contains datablock(s) I, global. DOI: 10.1107/S1600536811040475/bh2382sup1.cif

Structure factors: contains datablock(s) I. DOI: 10.1107/S1600536811040475/bh2382Isup2.hkl

Additional supplementary materials:  crystallographic information; 3D view; checkCIF report

## Figures and Tables

**Fig. 1.**
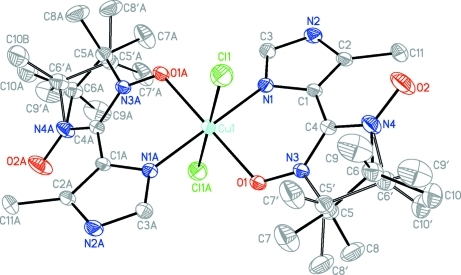
ORTEP drawing of title compound, showing 30% probability displacement ellipsoids. (H atoms are omitted for clarity) [symmetry code A: -x, -y, -z +1]. All disordered atoms are present.

**Fig. 2.**
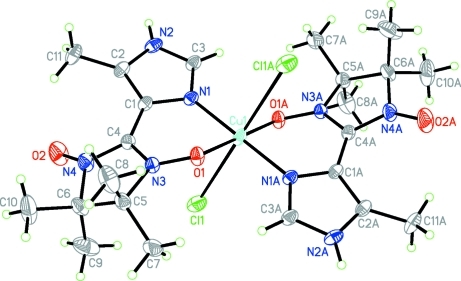
ORTEP drawing of the title compound omitting one set of disordered sites. Other features are as in Fig. 1.

## supplementary crystallographic information

### Comment

The design and synthesis of molecule-based magnetic materials is one of the major subjects of material sciences (Stroh *et al.*, 2003; Kahn *et al.*, 2000; Fursova *et al.*, 2003). In this field, nitronyl nitroxides acting as useful paramagnetic building blocks have been extensively used to assemble molecular magnetic materials due to their donor atoms and their ability to assemble extended arrangement with changing magnetic coupling (Muppidi *et al.*, 2006; Wang *et al.*, 2005; Gao *et al.*, 2010). In this article, we report the synthesis and crystal structure of a novel Cu^II^ complex with nitronyl nitroxide radical.

The crystal structure of the title complex is shown in Figures 1 and 2. The copper(II) ion is six-coordinated in a slightly distorted octahedral CuN_2_O_2_Cl_2_ environment. Two nitronyl nitroxide radicals, acting as bidentate chelating ligands, coordinate the Cu^II^ ion leading to two six-membered chelate rings, and the copper coordination is completed by two *trans*-arranged chloride anions.

### Experimental

The nitronyl nitroxide radical 4,4,5,5-tetramethyl-2-(5-methylimidazol-4-yl)-2-imidazoline-1-oxyl-3-oxide was prepared according to the literature method (Ullman *et al.*, 1970, 1972). The complex was synthesized by mixing 3 ml of an ethanol solution of the radical ligand (0.6 mmol) and 5 ml of an ethanol solution of CuCl_2_.2H_2_O (0.3 mmol). After stirring for two hours at room temperature, the mixture solution was filtered. The clear deep purple filtrate was diffused with diethyl ether vapor at room temperature. Deep purple crystals suitable for X-ray analysis were obtained after a week.

### Refinement

All H atoms attached to C atoms were geometrically fixed and allowed to ride on the corresponding parent atom with C—H = 0.96 Å and *U*_iso_(H) = 1.5*U*_eq_(C) for methyl groups or *U*_iso_(H) = 1.2*U*_eq_(C) for other H atoms. Two C atoms in one cycle and the four methyl groups bonded to these atoms (C5, C6, C7, C8, C9, C10) are disordered over two sites. Site occupancies were refined and converged to 0.737 (5) and 0.263 (5). The pair of C—C, C—N 1,2-distances and corresponding 1,3-distances were restrained to be equal within 0.01 Å of each other. The displacement parameters for C5 and C5', C6 and C6', C7 and C7', C8 and C8', C9 and C9', C10 and C10', were constrained to be the same using the *EADP* constraint (Sheldrick, 2008).

### Crystal data

**Table:**

[CuCl_2_(C_11_H_17_N_4_O_2_)_2_]	*F*(000) = 634
*M**_r_* = 609.01	*D*_x_ = 1.382 Mg m^−^^3^
Monoclinic, *P*2_1_/*c*	Mo *K*α radiation, λ = 0.71073 Å
Hall symbol: -P 2ybc	Cell parameters from 5379 reflections
*a* = 7.9966 (10) Å	θ = 2.6–28.0°
*b* = 14.1981 (18) Å	µ = 0.97 mm^−^^1^
*c* = 12.9266 (17) Å	*T* = 295 K
β = 94.169 (3)°	Block, deep purple
*V* = 1463.8 (3) Å^3^	0.43 × 0.27 × 0.17 mm
*Z* = 2	

### Data collection

**Table:**

Bruker SMART APEXII CCD area-detector diffractometer	3359 independent reflections
Radiation source: fine-focus sealed tube	2607 reflections with *I* > 2σ(*I*)
graphite	*R*_int_ = 0.027
φ and ω scans	θ_max_ = 27.5°, θ_min_ = 2.6°
Absorption correction: multi-scan (*SADABS*; Bruker, 2002)	*h* = −10→10
*T*_min_ = 0.682, *T*_max_ = 0.855	*k* = −18→18
12447 measured reflections	*l* = −16→16

### Refinement

**Table:**

Refinement on *F*^2^	Primary atom site location: structure-invariant direct methods
Least-squares matrix: full	Secondary atom site location: difference Fourier map
*R*[*F*^2^ > 2σ(*F*^2^)] = 0.060	Hydrogen site location: inferred from neighbouring sites
*wR*(*F*^2^) = 0.202	H-atom parameters constrained
*S* = 1.06	*w* = 1/[σ^2^(*F*_o_^2^) + (0.116*P*)^2^ + 1.4099*P*] where *P* = (*F*_o_^2^ + 2*F*_c_^2^)/3
3359 reflections	(Δ/σ)_max_ < 0.001
138 parameters	Δρ_max_ = 1.09 e Å^−^^3^
63 restraints	Δρ_min_ = −1.07 e Å^−^^3^

### Fractional atomic coordinates and isotropic or equivalent isotropic displacement parameters (Å^2^)

**Table:**

	*x*	*y*	*z*	*U*_iso_*/*U*_eq_	Occ. (<1)
C5	0.0161 (3)	0.2896 (2)	0.4252 (2)	0.0550 (10)	0.737 (5)
C6	0.1823 (3)	0.3180 (2)	0.3766 (3)	0.0604 (15)	0.737 (5)
C7	−0.1329 (4)	0.2749 (4)	0.3473 (4)	0.093 (3)	0.737 (5)
H7A	−0.1087	0.2248	0.3008	0.140*	0.737 (5)
H7B	−0.1545	0.3318	0.3085	0.140*	0.737 (5)
H7C	−0.2297	0.2587	0.3834	0.140*	0.737 (5)
C8	−0.0353 (6)	0.3521 (3)	0.5121 (3)	0.086 (2)	0.737 (5)
H8A	−0.1245	0.3225	0.5460	0.130*	0.737 (5)
H8B	−0.0730	0.4117	0.4843	0.130*	0.737 (5)
H8C	0.0589	0.3617	0.5614	0.130*	0.737 (5)
C9	0.1879 (7)	0.2920 (4)	0.2629 (3)	0.107 (3)	0.737 (5)
H9A	0.3007	0.2977	0.2430	0.161*	0.737 (5)
H9B	0.1160	0.3335	0.2214	0.161*	0.737 (5)
H9C	0.1503	0.2282	0.2526	0.161*	0.737 (5)
C10	0.2323 (6)	0.4199 (3)	0.3906 (5)	0.108 (3)	0.737 (5)
H10A	0.2538	0.4333	0.4632	0.162*	0.737 (5)
H10B	0.1431	0.4596	0.3622	0.162*	0.737 (5)
H10C	0.3317	0.4319	0.3553	0.162*	0.737 (5)
C5'	0.02011 (18)	0.28092 (9)	0.40266 (12)	0.0550 (10)	0.263 (5)
C6'	0.1900 (2)	0.33754 (9)	0.41609 (13)	0.0604 (15)	0.263 (5)
C7'	−0.02440 (19)	0.24924 (10)	0.29180 (12)	0.093 (3)	0.263 (5)
H7'1	−0.0396	0.1822	0.2903	0.140*	0.263 (5)
H7'2	0.0646	0.2660	0.2493	0.140*	0.263 (5)
H7'3	−0.1263	0.2795	0.2657	0.140*	0.263 (5)
C8'	−0.1284 (2)	0.33251 (12)	0.44123 (15)	0.086 (2)	0.263 (5)
H8'1	−0.2221	0.2903	0.4420	0.130*	0.263 (5)
H8'2	−0.1574	0.3846	0.3960	0.130*	0.263 (5)
H8'3	−0.1002	0.3555	0.5101	0.130*	0.263 (5)
C9'	0.2417 (3)	0.38454 (12)	0.31841 (15)	0.107 (3)	0.263 (5)
H9'1	0.3429	0.4198	0.3339	0.161*	0.263 (5)
H9'2	0.1543	0.4262	0.2919	0.161*	0.263 (5)
H9'3	0.2610	0.3374	0.2674	0.161*	0.263 (5)
C10'	0.2082 (2)	0.40044 (10)	0.51042 (16)	0.108 (3)	0.263 (5)
H10D	0.2993	0.3784	0.5566	0.162*	0.263 (5)
H10E	0.1063	0.3991	0.5453	0.162*	0.263 (5)
H10F	0.2305	0.4638	0.4893	0.162*	0.263 (5)
Cu1	0.00000 (14)	0.00000 (9)	0.50000 (9)	0.0509 (3)	
Cl1	0.13233 (14)	0.02450 (8)	0.29756 (8)	0.0751 (4)	
O1	−0.04541 (13)	0.13931 (9)	0.50405 (9)	0.0500 (6)	
O2	0.46321 (17)	0.25885 (10)	0.42078 (12)	0.0957 (12)	
C1	0.31257 (11)	0.10914 (7)	0.54255 (7)	0.0410 (7)	
C2	0.46835 (14)	0.10640 (9)	0.59493 (10)	0.0482 (8)	
C3	0.34150 (18)	−0.02968 (9)	0.60589 (10)	0.0512 (8)	
H3	0.3225	−0.0923	0.6225	0.061*	
C4	0.23128 (11)	0.18575 (6)	0.48583 (8)	0.0423 (7)	
C11	0.60017 (17)	0.17812 (12)	0.62182 (14)	0.0716 (12)	
H11A	0.5963	0.1960	0.6932	0.107*	
H11B	0.5809	0.2325	0.5785	0.107*	
H11C	0.7084	0.1521	0.6110	0.107*	
N1	0.23258 (13)	0.02450 (7)	0.55218 (8)	0.0426 (6)	
N2	0.48285 (17)	0.01743 (10)	0.63320 (11)	0.0541 (8)	
H2	0.5686	−0.0049	0.6690	0.065*	
N3	0.06688 (12)	0.19728 (7)	0.47178 (9)	0.0416 (6)	
N4	0.30710 (15)	0.25572 (8)	0.43551 (10)	0.0597 (8)	

### Atomic displacement parameters (Å^2^)

**Table:**

	*U*^11^	*U*^22^	*U*^33^	*U*^12^	*U*^13^	*U*^23^
C5	0.0462 (19)	0.047 (2)	0.071 (3)	0.0043 (16)	0.0012 (18)	0.016 (2)
C6	0.054 (2)	0.061 (3)	0.068 (4)	0.004 (2)	0.018 (2)	0.021 (3)
C7	0.064 (4)	0.103 (5)	0.109 (5)	−0.009 (4)	−0.024 (3)	0.058 (5)
C8	0.098 (5)	0.047 (3)	0.120 (6)	0.018 (3)	0.044 (4)	0.005 (3)
C9	0.105 (6)	0.152 (8)	0.069 (4)	0.021 (6)	0.026 (4)	0.039 (5)
C10	0.078 (4)	0.055 (4)	0.192 (10)	−0.011 (3)	0.017 (5)	0.020 (5)
C5'	0.0462 (19)	0.047 (2)	0.071 (3)	0.0043 (16)	0.0012 (18)	0.016 (2)
C6'	0.054 (2)	0.061 (3)	0.068 (4)	0.004 (2)	0.018 (2)	0.021 (3)
C7'	0.064 (4)	0.103 (5)	0.109 (5)	−0.009 (4)	−0.024 (3)	0.058 (5)
C8'	0.098 (5)	0.047 (3)	0.120 (6)	0.018 (3)	0.044 (4)	0.005 (3)
C9'	0.105 (6)	0.152 (8)	0.069 (4)	0.021 (6)	0.026 (4)	0.039 (5)
C10'	0.078 (4)	0.055 (4)	0.192 (10)	−0.011 (3)	0.017 (5)	0.020 (5)
Cu1	0.0331 (3)	0.0363 (3)	0.0801 (5)	0.0035 (2)	−0.0172 (3)	−0.0026 (3)
Cl1	0.0686 (7)	0.0770 (7)	0.0762 (7)	0.0088 (6)	−0.0188 (5)	−0.0067 (6)
O1	0.0322 (10)	0.0401 (13)	0.0783 (17)	0.0028 (9)	0.0084 (10)	0.0069 (11)
O2	0.0425 (16)	0.109 (3)	0.139 (3)	−0.0008 (17)	0.0273 (18)	0.035 (2)
C1	0.0298 (13)	0.0457 (17)	0.0472 (16)	0.0054 (12)	0.0004 (11)	−0.0064 (13)
C2	0.0319 (14)	0.056 (2)	0.0561 (19)	0.0057 (14)	0.0002 (13)	−0.0122 (15)
C3	0.0409 (18)	0.0487 (18)	0.062 (2)	0.0061 (15)	−0.0105 (15)	−0.0021 (16)
C4	0.0337 (14)	0.0451 (17)	0.0485 (17)	0.0016 (12)	0.0051 (12)	−0.0008 (13)
C11	0.0376 (18)	0.077 (3)	0.099 (3)	−0.0072 (19)	−0.0075 (19)	−0.019 (2)
N1	0.0354 (14)	0.0420 (13)	0.0493 (15)	0.0050 (11)	−0.0047 (11)	−0.0029 (11)
N2	0.0406 (15)	0.0618 (19)	0.0573 (17)	0.0129 (13)	−0.0132 (13)	−0.0074 (14)
N3	0.0324 (12)	0.0394 (14)	0.0533 (15)	0.0035 (10)	0.0057 (11)	0.0036 (11)
N4	0.0390 (15)	0.066 (2)	0.076 (2)	−0.0025 (14)	0.0132 (14)	0.0146 (17)

### Geometric parameters (Å, °)

**Table:**

C5—N3	1.487 (3)	C8'—H8'2	0.9600
C5—C8	1.5121	C8'—H8'3	0.9600
C5—C7	1.5170	C9'—H9'1	0.9600
C5—C6	1.5639	C9'—H9'2	0.9600
C6—N4	1.499 (3)	C9'—H9'3	0.9600
C6—C10	1.5086	C10'—H10D	0.9600
C6—C9	1.5189	C10'—H10E	0.9600
C7—H7A	0.9600	C10'—H10F	0.9600
C7—H7B	0.9600	Cu1—N1	1.9628
C7—H7C	0.9600	Cu1—N1^i^	1.963 (3)
C8—H8A	0.9600	Cu1—O1	2.0123
C8—H8B	0.9600	Cu1—O1^i^	2.012 (3)
C8—H8C	0.9600	Cu1—Cl1	2.9140
C9—H9A	0.9600	O1—N3	1.3086
C9—H9B	0.9600	O2—N4	1.2772
C9—H9C	0.9600	C1—N1	1.3713
C10—H10A	0.9600	C1—C2	1.3745
C10—H10B	0.9600	C1—C4	1.4401
C10—H10C	0.9600	C2—N2	1.3584
C5'—C8'	1.5106	C2—C11	1.4885
C5'—N3	1.5165	C3—N1	1.3208
C5'—C7'	1.5193	C3—N2	1.3385
C5'—C6'	1.5773	C3—H3	0.9300
C6'—N4	1.5016	C4—N3	1.3241
C6'—C10'	1.5099	C4—N4	1.3547
C6'—C9'	1.5121	C11—H11A	0.9600
C7'—H7'1	0.9600	C11—H11B	0.9600
C7'—H7'2	0.9600	C11—H11C	0.9600
C7'—H7'3	0.9600	N2—H2	0.8600
C8'—H8'1	0.9600		
			
N3—C5—C8	107.3 (2)	H10E—C10'—H10F	109.5
N3—C5—C7	109.0 (2)	N1—Cu1—N1^i^	180.00 (11)
C8—C5—C7	109.3	N1—Cu1—O1	89.1
N3—C5—C6	100.09 (17)	N1^i^—Cu1—O1	90.85 (11)
C8—C5—C6	115.6	N1—Cu1—O1^i^	90.85 (9)
C7—C5—C6	114.7	N1^i^—Cu1—O1^i^	89.15 (9)
N4—C6—C10	110.1 (2)	O1—Cu1—O1^i^	180.00 (13)
N4—C6—C9	106.4 (2)	N1—Cu1—Cl1	83.7
C10—C6—C9	108.9	N1^i^—Cu1—Cl1	96.28 (10)
N4—C6—C5	101.38 (18)	O1—Cu1—Cl1	89.1
C10—C6—C5	115.1	O1^i^—Cu1—Cl1	90.91 (10)
C9—C6—C5	114.3	N3—O1—Cu1	118.7
C8'—C5'—N3	110.6	N1—C1—C2	110.0
C8'—C5'—C7'	108.6	N1—C1—C4	120.8
N3—C5'—C7'	110.8	C2—C1—C4	129.2
C8'—C5'—C6'	114.1	N2—C2—C1	104.8
N3—C5'—C6'	99.2	N2—C2—C11	120.7
C7'—C5'—C6'	113.3	C1—C2—C11	134.2
N4—C6'—C10'	107.5	N1—C3—N2	111.1
N4—C6'—C9'	106.0	N1—C3—H3	124.5
C10'—C6'—C9'	113.4	N2—C3—H3	124.5
N4—C6'—C5'	98.4	N3—C4—N4	108.5
C10'—C6'—C5'	115.0	N3—C4—C1	124.8
C9'—C6'—C5'	114.8	N4—C4—C1	126.7
C5'—C7'—H7'1	109.5	C2—C11—H11A	109.5
C5'—C7'—H7'2	109.5	C2—C11—H11B	109.5
H7'1—C7'—H7'2	109.5	H11A—C11—H11B	109.5
C5'—C7'—H7'3	109.5	C2—C11—H11C	109.5
H7'1—C7'—H7'3	109.5	H11A—C11—H11C	109.5
H7'2—C7'—H7'3	109.5	H11B—C11—H11C	109.5
C5'—C8'—H8'1	109.5	C3—N1—C1	105.3
C5'—C8'—H8'2	109.5	C3—N1—Cu1	130.5
H8'1—C8'—H8'2	109.5	C1—N1—Cu1	124.1
C5'—C8'—H8'3	109.5	C3—N2—C2	108.7
H8'1—C8'—H8'3	109.5	C3—N2—H2	125.7
H8'2—C8'—H8'3	109.5	C2—N2—H2	125.7
C6'—C9'—H9'1	109.5	O1—N3—C4	125.2
C6'—C9'—H9'2	109.5	O1—N3—C5	120.67 (12)
H9'1—C9'—H9'2	109.5	C4—N3—C5	113.83 (12)
C6'—C9'—H9'3	109.5	O1—N3—C5'	122.3
H9'1—C9'—H9'3	109.5	C4—N3—C5'	112.2
H9'2—C9'—H9'3	109.5	O2—N4—C4	125.0
C6'—C10'—H10D	109.5	O2—N4—C6	121.70 (12)
C6'—C10'—H10E	109.5	C4—N4—C6	111.83 (12)
H10D—C10'—H10E	109.5	O2—N4—C6'	123.5
C6'—C10'—H10F	109.5	C4—N4—C6'	110.6
H10D—C10'—H10F	109.5		
			
N3—C5—C6—N4	−19.0 (2)	C1—C4—N3—O1	−2.6
C8—C5—C6—N4	95.9 (3)	N4—C4—N3—C5	−11.16 (16)
C7—C5—C6—N4	−135.5 (3)	C1—C4—N3—C5	170.75 (16)
N3—C5—C6—C10	−137.8 (2)	N4—C4—N3—C5'	2.0
C8—C5—C6—C10	−23.0	C1—C4—N3—C5'	−176.1
C7—C5—C6—C10	105.7	C8—C5—N3—O1	72.32 (16)
N3—C5—C6—C9	95.0 (2)	C7—C5—N3—O1	−45.98 (16)
C8—C5—C6—C9	−150.1	C6—C5—N3—O1	−166.68 (13)
C7—C5—C6—C9	−21.4	C8—C5—N3—C4	−101.31 (18)
C8'—C5'—C6'—N4	146.8	C7—C5—N3—C4	140.39 (17)
N3—C5'—C6'—N4	29.3	C6—C5—N3—C4	19.68 (19)
C7'—C5'—C6'—N4	−88.2	C8—C5—N3—C5'	173.9 (6)
C8'—C5'—C6'—C10'	33.0	C7—C5—N3—C5'	55.6 (5)
N3—C5'—C6'—C10'	−84.5	C6—C5—N3—C5'	−65.2 (5)
C7'—C5'—C6'—C10'	158.0	C8'—C5'—N3—O1	44.7
C8'—C5'—C6'—C9'	−101.1	C7'—C5'—N3—O1	−75.8
N3—C5'—C6'—C9'	141.3	C6'—C5'—N3—O1	164.9
C7'—C5'—C6'—C9'	23.8	C8'—C5'—N3—C4	−141.5
N1—Cu1—O1—N3	42.2	C7'—C5'—N3—C4	98.0
N1^i^—Cu1—O1—N3	−137.83 (10)	C6'—C5'—N3—C4	−21.3
Cl1—Cu1—O1—N3	−41.6	C8'—C5'—N3—C5	−41.2 (6)
N1—C1—C2—N2	2.1	C7'—C5'—N3—C5	−161.7 (6)
C4—C1—C2—N2	−179.4	C6'—C5'—N3—C5	79.0 (6)
N1—C1—C2—C11	−171.6	N3—C4—N4—O2	−169.8
C4—C1—C2—C11	6.9	C1—C4—N4—O2	8.3
N1—C1—C4—N3	25.7	N3—C4—N4—C6	−3.46 (17)
C2—C1—C4—N3	−152.7	C1—C4—N4—C6	174.59 (17)
N1—C1—C4—N4	−152.0	N3—C4—N4—C6'	20.5
C2—C1—C4—N4	29.5	C1—C4—N4—C6'	−161.5
N2—C3—N1—C1	2.0	C10—C6—N4—O2	−55.76 (15)
N2—C3—N1—Cu1	−174.8	C9—C6—N4—O2	62.09 (14)
C2—C1—N1—C3	−2.5	C5—C6—N4—O2	−178.12 (12)
C4—C1—N1—C3	178.8	C10—C6—N4—C4	137.42 (16)
C2—C1—N1—Cu1	174.6	C9—C6—N4—C4	−104.73 (17)
C4—C1—N1—Cu1	−4.1	C5—C6—N4—C4	15.1 (2)
O1—Cu1—N1—C3	153.7	C10—C6—N4—C6'	46.1 (3)
O1^i^—Cu1—N1—C3	−26.25 (10)	C9—C6—N4—C6'	163.9 (3)
Cl1—Cu1—N1—C3	−117.1	C5—C6—N4—C6'	−76.3 (3)
O1—Cu1—N1—C1	−22.6	C10'—C6'—N4—O2	−82.5
O1^i^—Cu1—N1—C1	157.41 (10)	C9'—C6'—N4—O2	39.1
Cl1—Cu1—N1—C1	66.6	C5'—C6'—N4—O2	157.9
N1—C3—N2—C2	−0.8	C10'—C6'—N4—C4	87.5
C1—C2—N2—C3	−0.8	C9'—C6'—N4—C4	−151.0
C11—C2—N2—C3	174.0	C5'—C6'—N4—C4	−32.1
Cu1—O1—N3—C4	−36.8	C10'—C6'—N4—C6	−175.1 (3)
Cu1—O1—N3—C5	150.33 (17)	C9'—C6'—N4—C6	−53.5 (3)
Cu1—O1—N3—C5'	136.2	C5'—C6'—N4—C6	65.3 (3)
N4—C4—N3—O1	175.5		

Symmetry codes: (i) −*x*, −*y*, −*z*+1.
